# Assessing Vaccine Intentions, Knowledge, Self-Efficacy, and Trust: A Cross-Sectional Study on Perceptions of Monkeypox Vaccination and Public Health Risk Awareness in Makurdi, Benue State, Nigeria

**DOI:** 10.7759/cureus.72131

**Published:** 2024-10-22

**Authors:** Adewale Lawrence

**Affiliations:** 1 Pharmaceutical Medicine, Bioluminux Clinical Research, Naperville, USA

**Keywords:** benue state, covid-19 vaccination, monkeypox, nigeria, public health, self-efficacy, trust in vaccines, vaccination intention

## Abstract

Introduction

The global emergence of monkeypox has heightened public health concerns, particularly in regions with limited vaccine uptake. In Nigeria, understanding the factors influencing vaccination intentions is essential for developing effective public health strategies. Despite vaccine availability, hesitancy and varying degrees of acceptance persist, driven by factors such as self-efficacy, vaccine trust, and overall attitudes toward vaccination. Previous research highlights the importance of these factors in shaping health behaviors, particularly in vulnerable populations. In Benue State, where distinct health challenges affect key demographic groups such as people living with human immunodeficiency virus (PLHIV) and men who have sex with men (MSM), addressing vaccination intentions is critical for public health efforts.

Objectives

The objective was to assess the intention to receive the monkeypox vaccine, self-assessed knowledge levels, self-efficacy, and risk perception among the PLHIV, MSM, and the healthcare population in Benue State, Nigeria. Additionally, the study aimed to evaluate public trust in vaccines and health authorities and to understand how these factors influence attitudes toward vaccination and overall perceptions of monkeypox-related health risks.

Methodology

A cross-sectional survey was conducted among 377 participants in Makurdi, Benue State, Nigeria, focusing on PLHIV, MSM, and healthcare workers. Participants were recruited using a snowball sampling technique, which leveraged social media platforms (WhatsApp, Facebook, and Telegram) and the Federal Medical Center in Makurdi. Data were collected using a structured questionnaire designed to assess self-efficacy, trust in vaccines, vaccination attitudes, and prior vaccination experiences. Statistical analysis was performed using IBM SPSS Statistics for Windows, version 25.0 (IBM Corp., Armonk, NY). Descriptive statistics were used to summarize the characteristics of the study population. Bivariate analyses, including chi-square tests and independent t-tests, were employed to explore the relationships between socio-demographic characteristics and vaccination intentions. Statistical significance was set at p < 0.05 for all analyses.

Results

The study revealed a significant positive relationship between self-efficacy and vaccination intention (p = 0.001). Trust in vaccinations and health authorities was also found to be a significant predictor of vaccination intention (p < 0.05). Furthermore, participants who had previously received the coronavirus disease 2019 (COVID-19) vaccine were more likely to express intention to receive the monkeypox vaccine (p = 0.000). However, the perception that the threat of monkeypox was exaggerated did not significantly impact vaccination intention (p = 0.4).

Conclusion

This study highlights the critical role of self-efficacy, trust in vaccines, and prior vaccination experiences in shaping the intention to receive the monkeypox vaccine in Benue State, Nigeria. The findings suggest that public health policies should prioritize enhancing trust in vaccines and health authorities, as well as boosting self-efficacy, to improve vaccination uptake. Addressing these factors may lead to more effective public health interventions against monkeypox and other infectious diseases.

## Introduction

Monkeypox is a re-emerging viral zoonotic disease caused by the Mpox virus (MPXV), which belongs to the *Orthopoxvirus* genus and the *Poxviridae* family [1]. *Orthopoxvirus*, MPXV, and smallpox viruses are zoonotic, making them the most important members of the genus in terms of public health and One Health [2]. Mpox has emerged attention as a public health problem due to its complex epidemiology and the increased danger it poses to vulnerable communities, particularly in Sub-Saharan Africa [3]. Recent outbreaks, particularly those observed in 2022, have highlighted concerning tendencies in transmission, particularly among people already disadvantaged by health inequities, such as those living with human immunodeficiency virus (HIV) [4]. Understanding people’s perspectives, knowledge, and risk awareness about Mpox transmission is critical for developing effective public health strategies [5].

An effective response to the human (MPXV) emergency necessitates tailored public health programs that address the requirements of all impacted areas [6]. As we have seen in both the HIV [7] and coronavirus disease 2019 (COVID-19) pandemics, the spread of disinformation and marginalization have posed substantial challenges to pandemic containment and continue to do so [8] due to delays in diagnosis, treatment, and vaccine adoption [9]. This has exacerbated health disparities among minoritized and structurally marginalized communities, particularly among those living with HIV, who have been disproportionately affected by the global outbreak. A global case series of 528 persons with MPXV reported that 41% were people living with HIV (PLHIV), and 57% of those without HIV received pre-exposure prophylaxis (PrEP) for HIV [10].

Historically, Mpox has been a chronic concern, although many people are unaware of its transmission dynamics and risks [11]. Misconceptions about the disease are prevalent, especially in areas with inadequate healthcare infrastructure and limited access to information. This lack of awareness may result in inadequate preventive methods, increasing vulnerability to infection [12]. Furthermore, the stigma associated with specific populations, such as men who have sex with men (MSM) and PLHIV, can hinder public health initiatives, leading to greater marginalization and limited access to care [13].

Public health information dissemination is critical for impacting community attitudes and responses to infectious disease epidemics [14]. Effective communication tactics can raise awareness, correct misinformation, and encourage proactive health habits [15]. This study aims to identify knowledge gaps and areas for improvement in communication efforts by evaluating the effectiveness of existing public health messaging.

The growing number of Mpox cases, as well as the special obstacles that at-risk communities confront [16], highlight the significance of our study. Tailored public health interventions that address these information gaps are critical to generating informed community responses and improving health outcomes. This research is not just intended to provide insights into public knowledge of Mpox while also contributing to the broader discussion of public health communication and its role in infectious disease management. By better understanding and tackling the stigma associated with Mpox and its transmission, we may better equip communities to respond to present and future health concerns. This study aims to address knowledge gaps and perceptions about monkeypox transmission, particularly among vulnerable populations. Recent outbreaks have highlighted a resurgence, particularly among high-risk groups like MSM and HIV-positive individuals [17]. Targeted public health campaigns should address specific community needs and behavioral risks. Understanding stigma and improving public health messaging can foster inclusive approaches. The findings will help develop tailored interventions, improve health outcomes, and reduce transmission rates.

Objectives

The objective of this study was to assess the intention to receive the monkeypox vaccine, self-assessed knowledge levels, self-efficacy, and risk perception among the general population in Benue State, Nigeria. Additionally, the study aimed to evaluate public trust in vaccines and health authorities and to understand how these factors influence attitudes toward vaccination and overall perceptions of monkeypox-related health risks.

## Materials and methods

Study design and setting 

This cross-sectional study was conducted in Makurdi, Benue State, North-Central Nigeria, targeting PLHIV, MSM, and healthcare workers. A snowball sampling strategy was employed to distribute an online survey via Google Forms. Potential participants were recruited through the Federal Medical Center in Makurdi and social media platforms (WhatsApp, Facebook, and Telegram) after being informed about the study’s objectives. Participants who consented to join the study were given the option to complete a self-administered questionnaire either on paper or online.

Study duration and date of institutional review board approval

The study was conducted between July and September 2024 after the institutional review board letter was approved by The Health Research Ethics Committee on July 8, 2024.

Sample size calculation

**Figure 1:**
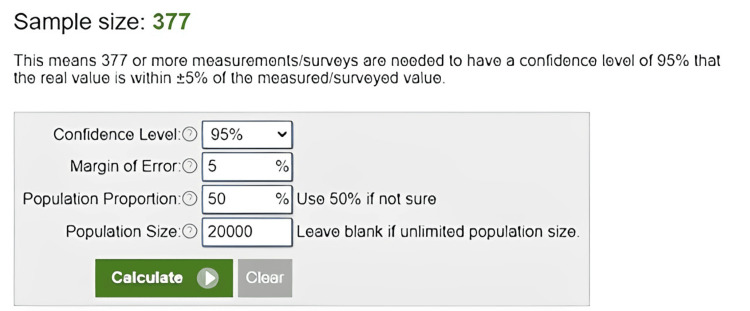
Sample size calculation details

The sample size was calculated using Raosoft software, with a minimum required sample size of 377 (Figure 1).

This ensures a 95% confidence level that the true population value lies within ±5% of the measured or surveyed value.

Eligibility criteria

Inclusion Criteria

Participants included adults aged 18 years and older, specifically PLHIV, MSM, individuals without cognitive impairments, and healthcare workers recruited from the Federal Medical Center and affiliate community clinics in Makurdi. MSM individuals residing in the Makurdi metropolis who consented to participate were also included.

Exclusion Criteria

Individuals excluded from the study were those who were unable to provide consent due to ill health.

The online survey questionnaire assessed respondents’ awareness of monkeypox symptoms, knowledge, risk perceptions, and self-efficacy. It also explored trusted information sources, preferred stakeholders for outbreak response, vaccine trust indicators (VTIs), and intentions regarding a monkeypox vaccine. Key measures included self-assessed knowledge levels, a composite risk perception variable, self-efficacy, and the VTI [18].

Data were collected using a self-administered questionnaire in English, divided into three sections: section one consists of socio-demographic information; section two assesses the self-assessed level of knowledge, self-efficacy, and intentions to receive the Mpox vaccine; and section three comprises trust in vaccine manufacturers or pharmaceutical companies, trust in the Department of Health Services, understanding of vaccination benefits, importance of getting vaccinated, and vaccination as part of a healthy lifestyle.

To ensure inclusivity, both online and hard-copy versions of the questionnaire were made available. The online survey was hosted on platforms such as Google Forms, while hard copies were distributed through community hospitals, healthcare facilities, and local support groups for participants lacking reliable internet access. Trained personnel facilitated the distribution and collection of hard-copy questionnaires, and data from the hard copies were manually entered into the online platform to create a consolidated dataset for analysis.

Statistical analysis plan

Data were reviewed for completeness, accuracy, and consistency prior to analysis, and any missing values were addressed appropriately. Chi-square tests were utilized to examine associations between socio-demographic variables and the intention to receive the monkeypox vaccine. All statistical analyses were performed using IBM SPSS Statistics for Windows, version 25.0 (IBM Corp., Armonk, NY), with statistical significance set at a p-value of < 0.05.

Ethical consideration

This study adhered to the principles outlined in the Declaration of Helsinki and received approval from The Health Research Ethics Committee Institutional Review Board under reference number FMH/FMC/HREC/108/VOL.I. Informed consent was obtained from all participants prior to their involvement in the study. Participation was voluntary, and participants had the right to withdraw at any time without consequence.

Confidentiality was strictly maintained, with participants’ data anonymized to ensure privacy. The study was conducted with integrity, transparency, and full respect for participants’ rights and welfare. Efforts were made to minimize any potential risks, ensuring that the benefits of the research outweighed any possible harms. All conflicts of interest were disclosed, and the research complied with relevant laws, regulations, and ethical standards throughout its duration.

## Results

Demographic characteristics of the participants

Table 1 summarizes the demographic characteristics of the study participants. The demographic summary shows that the majority of participants were female (63.9%), with males making up 35.8% and a small minority identifying as “other” (0.3%). Most respondents (74.8%) were aged between 30 and 60, with 20.4% aged 18-29, and 4.8% over 60. Nearly all participants (99.7%) were Nigerian, and 62.1% were married. A majority (68.2%) lived in cities, and 65.8% were from Benue State. Occupationally, 43.5% were physicians or physician assistants, while 53.8% had no formal education. Further details can be found in the table.

**Table 1:** Demographic characteristics of the participants

Variables	Category	Frequency (n = 377)	Percent (100%)
Gender	Male	135	35.8
	Female	241	63.9
	Others	1	0.3
Age	18-29 years	77	20.4
	30-60 years	282	74.8
	Above 60 years	18	4.8
Nationality	Nigerian	376	99.7
	Other	1	0.3
Martial status	Single	85	22.5
	Married	234	62.1
	Having partner	10	2.7
	Widow	46	12.2
	Other	2	0.5
Region	Benue State	250	65.8
	Other	126	32.6
Occupation	Physician/physician assistant	164	43.5
	Nurse/midwife	62	16.4
	Pharmacist/pharmacy technician	20	5.3
	Laboratory technician	130	34.5

Cross-tab analysis of the association between self-efficacy levels and intention to get vaccinated against monkeypox

Table 2 presents the distribution of self-efficacy levels among participants based on their intention to receive the monkeypox vaccine if recommended. Participants were categorized into three self-efficacy groups: no self-efficacy, neutral, and self-efficacy. Vaccination intentions were divided into two categories: “yes” (indicating willingness to be vaccinated) and “no/I don’t know” (indicating uncertainty or unwillingness). The p-value for the association between self-efficacy and vaccination intention is 0.001, indicating a statistically significant relationship.

**Table 2:** Cross-tabulation of self-efficacy The chi-square test was used to determine the association between self-efficacy and intention to get vaccinated against monkeypox. P-values were considered significant at p < 0.05 and highly significant at p < 0.001. An asterisk “*” indicates statistical significance (p <0.05).

Variable	Category		No self-efficacy	Neutral	Self-efficacy	Total	p-value
Intention to get vaccinated against monkeypox, if recommended		Yes	29	95	174	298	0.001*
		No, I don't know	9	41	27	77	
Total			38	136	201	375	

Risk perception

Chi-square tests were used to analyze associations between intention to receive monkeypox vaccination and the following factors: perceived exaggeration of the threat, vaccine trust indicator, and COVID-19 vaccination status. P-values were considered significant at p < 0.05 and highly significant at p < 0.001. For perceived exaggeration of the threat, no significant association was found (p = 0.4). However, vaccine trust indicator (p = 0.001) and COVID-19 vaccination status (p = 0.000) showed significant associations with the intention to receive monkeypox vaccination. In Table 3, the results are explained in detail.

**Table 3:** Factors influencing intention to receive monkeypox vaccination An asterisk “*” indicates statistical significance (p <0.05).

Variable			Category		No exaggeration	Neutral	Exaggeration	Total	p-value
Intention to get vaccinated against monkeypox, if recommended			Yes		152	101	47	300	0.4
			No, I don't know		40	29	8	77	
Total					192	130	55	377	
Vaccine trust indicator									
Variable				Category	Low	Medium	High	Total	p-value
Intention to get vaccinated against monkeypox, if recommended				Yes	38	137	125	300	0.001*
				No, I don't know	23	34	20	77	
Total					61	171	145	377	
COVID-19 vaccination status									
Variable	Category				Not vaccinated	Vaccinated	Total		p-value
Intention to get vaccinated against monkeypox, if recommended		Yes			107	193	300		0.000*
		No, I don't know			47	30	77		
Total					154	223	377		

Cross-tabulation of general vaccination attitudes and trust factors influencing monkeypox vaccination intentions

Table 4 investigates the factors influencing participants’ intentions to receive the monkeypox vaccine, focusing on overall vaccination attitudes, trust in health authorities and vaccine manufacturers, comprehension of vaccination benefits, and the perceived importance of vaccination. Significant associations were found between these variables and the desire to get vaccinated, indicating that positive vaccination attitudes and trust are key determinants of participants’ willingness to accept the monkeypox vaccine.

Table 4 explains that chi-square tests were used to analyze associations between general vaccination attitudes, trust factors, and monkeypox vaccination intentions. P-values were considered significant at p < 0.05 and highly significant at p < 0.001. Significant associations were found between monkeypox vaccination intention and being strongly for or against vaccination in general (p = 0.03), trust in the Department of Health Services (p = 0.03), trust in vaccine manufacturers (p = 0.03), understanding how vaccination helps the body with infectious diseases (p = 0.01), and feeling it is important to get vaccinated (p = 0.00).

**Table 4:** General vaccination attitudes and trust factors influencing monkeypox vaccination intentions An asterisk “*” indicates statistical significance (p < 0.05).

Variable	Category	Strongly against vaccination	Strongly for vaccination	Total	p-value
Intention to get vaccinated against monkeypox, if recommended	Yes	7	293	300	0.03*
	No, I don't know	8	67	75	
Total		15	360	375	
I generally trust the Department of Health Services					
Intention to get vaccinated against monkeypox, if recommended	Yes	5	295	300	0.03*
	No, I don't know	4	71	75	
Total		9	366	375	
I generally trust vaccine manufacturers or pharmaceutical companies					
Intention to get vaccinated against monkeypox, if recommended	Yes	11	289	300	0.03*
	No, I don't know	10	65	75	
Total		21	354	375	
I understand how vaccination helps my body with infectious diseases					
Intention to get vaccinated against monkeypox, if recommended	Yes	5	294	299	0.01*
	No, I don't know	8	69	77	
Total		13	363	376	
I feel it is important that I get vaccinated					
Intention to get vaccinated against monkeypox, if recommended	Yes	7	292	299	0.00*
	No, I don't know	10	67	77	
Total		17	359	376	

## Discussion

This study evaluated the factors influencing vaccination intentions against monkeypox across several demographic groups in Benue State, Nigeria, with a focus on self-efficacy, trust in vaccines, general vaccination attitudes, and prior COVID-19 vaccination status. The results revealed a significant correlation (p < 0.05) between vaccination intention and self-efficacy, trust in vaccines, general attitudes toward vaccination, and trust in the Department of Health Services, emphasizing the crucial role of trust and self-confidence in health-related decision-making.

Participants with greater confidence in their health decision-making abilities were significantly more likely to intend to vaccinate against monkeypox (p = 0.001), underlining the importance of empowering individuals. These findings are consistent with a study by Bish et al. (2011), which found that individuals with stronger self-efficacy were more likely to receive vaccines and recommend them to others [19]. Similarly, Dempsey et al. (2006) found that parents with higher self-efficacy were more likely to complete the human papillomavirus vaccine series for their children, emphasizing the relevance of self-efficacy in diverse health behaviors [20]. Wong et al. (2020) also demonstrated that higher self-efficacy correlates with increased engagement in preventive health behaviors, including vaccination [21].

Trust in vaccines and health authorities emerged as a strong predictor of vaccination intention, a phenomenon well-documented in the literature. Larson et al. (2018) emphasized that trust in healthcare institutions and vaccine manufacturers is a critical driver of vaccine acceptance [22]. In this study, participants with strong trust in vaccines were significantly more likely to receive the monkeypox vaccine (p = 0.001). Additionally, trust in the Department of Health Services (p = 0.03) and vaccine manufacturers (p = 0.03) positively influenced vaccination intentions. These findings align with Fisher et al. (2020), who emphasized the importance of endorsement and transparency from health institutions in shaping vaccine behaviors [23].

Moreover, Quinn et al. (2019) demonstrated that trust in public health agencies and healthcare providers was a significant predictor of influenza vaccine uptake, particularly in minority communities that may have historical skepticism toward public health interventions [24]. The SAGE Working Group on Vaccine Hesitancy (2014) similarly identified trust as one of the key determinants of vaccine acceptance globally, with low trust correlating with increased vaccine hesitancy [25]. Betsch et al. (2016) further underscored that trust in public health authorities enhances the perceived benefits of vaccination, thereby increasing vaccination intentions. This is consistent with our findings and highlights the need to build trust in health institutions [26].

Our study also found that prior COVID-19 vaccination status significantly influenced monkeypox vaccine acceptance (p = 0.000). Participants who had previously received the COVID-19 vaccine were more inclined to consider the monkeypox vaccine, a finding consistent with Yaqub et al. (2014), who noted that individuals with positive views toward vaccination are more likely to support repeated vaccinations [27].

Interestingly, contrary to expectations, the perceived exaggeration of the monkeypox threat did not significantly impact vaccination intention (p = 0.4). This finding contrasts with studies like Savoia et al. (2021) on COVID-19, where perceived severity was a strong determinant of vaccine uptake. However, lower perceptions of monkeypox severity may not reduce vaccination intentions, provided there is strong trust in the health system, high self-efficacy, and positive past vaccination experiences [28].

These findings have several implications for public health strategies. First, enhancing self-efficacy through targeted educational campaigns that provide clear, actionable information about monkeypox transmission and vaccination could significantly improve vaccine uptake. Second, building and maintaining trust in health authorities and vaccine manufacturers is critical, especially in regions where misinformation and skepticism are prevalent. Efforts to improve transparency, responsiveness, and engagement in health communication can help address these challenges and foster long-term trust.

Recommendations

Enhance Education and Awareness

Develop tailored educational campaigns to boost self-efficacy regarding monkeypox vaccination. These campaigns should provide clear, actionable information about the disease, its transmission, and the benefits of vaccination.

Leverage Past Vaccination Experiences

Utilize the positive reception of COVID-19 vaccinations as a platform to promote monkeypox vaccination. Public health messages should highlight similarities between the vaccines and emphasize the importance of continued vaccination efforts.

Community Engagement

Encourage community involvement in vaccination programs to promote culturally responsive approaches that address vaccine hesitancy and reluctance.

Monitor and Address Misinformation

Actively monitor and address misinformation about monkeypox and vaccines on social media and in community channels. Establish a rapid response team to counter false information and provide reassurance to the public.

Expand Future Research

Conduct further studies to explore additional factors influencing vaccination intentions, particularly among groups with a history of skepticism toward vaccinations.

Limitations

Self-Reported Data

Self-reported data on immunization intentions and prior actions may be subject to bias, as participants might provide socially desirable responses.

Limited Exploration of External Influences

The study primarily focused on individual determinants, potentially overlooking external factors such as socio-political conditions, health infrastructure, and community dynamics, which could influence vaccination intentions.

Perception Measurement

The perception of monkeypox as a threat was not significantly linked to vaccination intention, indicating that other contextual factors may need to be explored in future research to better understand vaccination behaviors.

## Conclusions

The study’s findings revealed that a range of psychological, perceptual, and trust-related factors strongly influenced the intention to get vaccinated against monkeypox among high-risk populations, including PLHIV, MSM, and healthcare workers. The strongest predictors of vaccination intention were self-efficacy, trust in vaccines, past COVID-19 vaccination status, and overall attitudes toward vaccination. High self-efficacy and strong trust in vaccines and public health authorities were significantly associated with a greater intention to get vaccinated, highlighting the need to build confidence and trust in vaccination programs. However, the perceived amplification of the monkeypox threat had no significant effect, indicating that threat perception alone was insufficient to alter vaccination intentions.

These findings underscore the importance of targeted public health interventions aimed at enhancing vaccine confidence, improving public trust in health institutions, and delivering clear educational messages that promote understanding of vaccine benefits. Such efforts could play a vital role in increasing monkeypox vaccination rates, reducing the risk of infection, and improving health outcomes in these vulnerable communities.

## Appendices

QUESTIONNAIRE __ Healthcare staff administered

Monkeypox is a disease that is spreading worldwide, and we, doctors and researchers from Bioluminux Clinical United States and the Federal University of Health Sciences Otukpo, Benue State, Nigeria, invite you to participate in this study. The study aims to assess the psychological motives for vaccinations against monkeypox.

Consent question:

1. Participate in the survey

a) Agree

b) Disagree

Section 1: *Socio-demographics questions*

2. Gender

a) Male

b) Female

c) Other

3. Age in years ………….

4. Occupation

a) Physician/physician assistant

b) Nurse/midwife

c) Pharmacist/pharmacy technician

d) Laboratory technician

e) Public health personnel

5. Region

Indicators

1. Intention to get vaccinated against monkeypox, if recommended

a) No/I don’t know

b) Yes

2. Self-assessed level of knowledge

a) Poor/very poor

b) Average

c) Good/very good

3. Self-efficacy

a) No self-efficacy

b) Neutral

c) Self-efficacy

Risk perception (mean)

4. Perceived exaggeration of threat

a) No exaggeration

b) Neutral

c) Exaggeration

5. Vaccine trust indicator

a) Low

b) Medium

c) High

6. COVID-19 vaccination status

a) Not vaccinated

b) Vaccinated

Vaccine Trust Indicator Item

7. Thinking about vaccination in general, would you say you are personally:

a) 0: strongly against vaccination

b) 10: strongly for vaccination

8. I generally trust vaccine manufacturers or pharmaceutical companies

a) 0: strongly against vaccination

b) 10: strongly for vaccination

9. I generally trust the Department of Health Services

a) 0: strongly against vaccination

b) 10: strongly for vaccination

10. I understand how vaccination helps my body fight infectious disease

a) 0: strongly against vaccination

b) 10: strongly for vaccination

11. I feel it is important that I get vaccinated

a) 0: strongly against vaccination

b) 10: strongly for vaccination

12. Vaccination forms part of a healthy lifestyle

a) 0: strongly against vaccination

b) 10: strongly for vaccination

Thank you for your participation. Link of the Study ( https://journals.plos.org/plosone/article?id=10.1371/journal.pone.0278622) The reference to the study is mentioned in the main text as well [15].
